# An uncommon long-term survival case of primary cardiac leiomyosarcoma

**DOI:** 10.1186/1477-7819-12-338

**Published:** 2014-11-10

**Authors:** Meriam Glaoui, Zineb Benbrahim, Rhizlane Belbaraka, Sara Naciri, Hassan Errihani, Axel Lescene

**Affiliations:** Department of Medical Oncology, clinique le littoral, salaj 3, Ain diab, Casablanca, Morocco; Gustave Roussy Institute, 114 Rue Édouard Vaillant, 94805 Villejuif, France; Department of medical oncology, Mohamed VI University hospital, Faculty of medicine of Marrakesh, Cadi ayad University, BP7010, Sidi Abbad, Marrakesh Morocco; Department of Medical Oncology, National institute of oncology, hay irfane, 10100 Rabat, Morocco

**Keywords:** Cardiac neoplasm, Leiomyosarcoma, Survival

## Abstract

Primary cardiac sarcoma is a rare aggressive entity. It constitutes the second most common type of primary cardiac neoplasms. Its management has largely been guided by small retrospective series with a median survival of 6 months. Here, we discuss a unique case of 8-year survival cardiac leiomyosarcoma managed by surgical and adjuvant therapy.

## Background

Primary cardiac sarcomas constitute a rare entity. At present, only a few hundred primary cardiac sarcomas have been reported, most of which are based on autopsy series. Despite this rarity, they are the second most common type of primary cardiac neoplasm and account for most of the malignant primary cardiac tumors. Primary cardiac sarcomas are aggressive tumors that generally do not produce symptoms until they are locally advanced. Their management has largely been guided by small retrospective series and non-cardiac sarcoma management principles. However, the prognosis of primary cardiac sarcomas in general remains poor according to the current medical literature. The median survival in patients with cardiac sarcomas has been reported to be about 6 months with a mean of 11 months. Here, we discuss a unique case of 8-year survival cardiac leiomyosarcoma managed by surgical and adjuvant therapy.

## Case presentation

**Figure 1:**
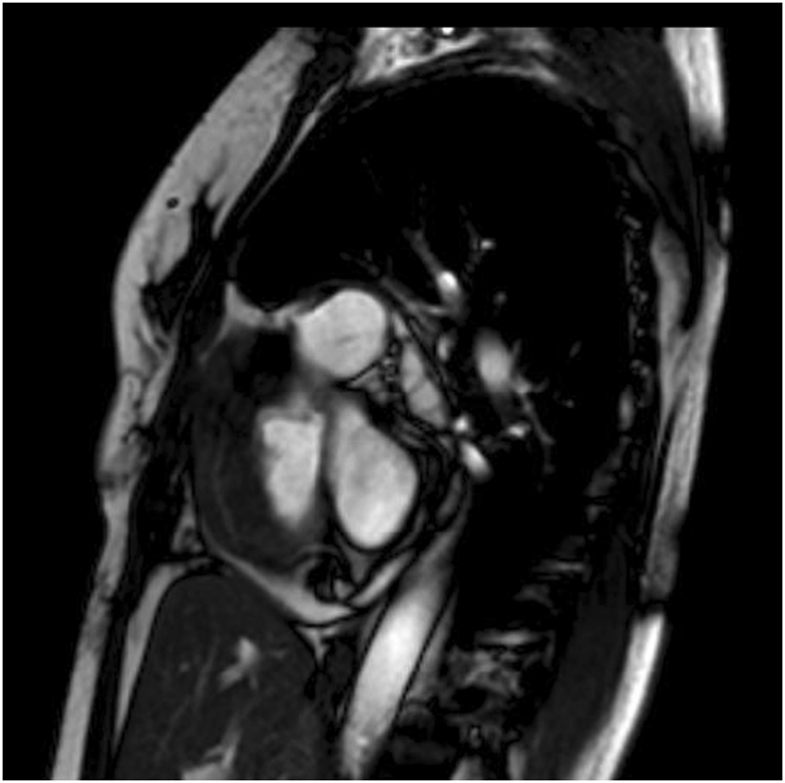
**MRI T2 cuts, minor axis passing throughout the right atrium showing an irregular isosignal wall thickening of the side wall of the right atrium.**

A 47-year-old man presented with acute pulmonary edema with severe progressive dyspnea and orthopnea. He had a 10 pack-year history of cigarette smoking, but no other medical or familial conditions. Chest radiograph showed a small right-sided pleural effusion. Electrocardiogram revealed sinus tachycardia without any conduction abnormalities. Transthoracic echocardiography and cardiac magnetic resonance imaging (CRMI) showed a tumor (4 × 5 cm) occupying the side wall of the right atrium budding into the lumen (Figure 1).

Through a median sternotomy, a radical excision of the neoplasm was performed; the atrial wall was reconstructed using a bovine pericardial patch. The post-operative course was unremarkable and the anatomical pathology analysis revealed fusiform cells with considerable nuclear pleomorphism and mitotic activity. The immunohistochemical study was positive to vimentin, desmin, actin, and HHF-35, and negative for PS-100, cytokeratin, and hormone receptors. The final diagnosis was cardiac leiomyosarcoma. Adjuvant chemotherapy with doxorubicin (60 mg/m^2^ day 1) and ifosfamide (3 g/m^2^ day 1 and 2) were realized and MRI after the four cycles did not show any sign of local or distant relapse.

Seven years after the first operation, local recurrence with a metastatic paravertebral soft tissue mass was revealed by magnetic resonance imaging. There was no lung metastasis. The patient received antalgic vertebral radiotherapy with systemic palliative chemotherapy consisting of oral cyclophosphamide. One year later, he died of progressive disease and multiorgan failure.

## Conclusions

Primary leiomyosarcomas of the heart [1] are extremely rare, accounting for 0.019% of all malignant cardiac neoplasms in autopsy studies [2]. They typically occur in the fourth decade with no sex predilection. As all other cardiac sarcomas, primary leiomyosarcoma of the heart are usually asymptomatic until an advanced stage. Consequently, the diagnosis is often clinically delayed until hemodynamic consequences and embolisms. Cardiac MRI is the gold standard complementary exam to distinguish malignant lesions and assess the resectability of the tumors.

Due to the small series reporting this disease, the treatment guidelines are not well defined. Radical surgical interventions seem to offer the best outcome; however, a complete surgical resection is often difficult to achieve. The role of adjuvant chemotherapy and radiotherapy to prolong survival in this group of patients is not clear [3–5]. Another therapeutic option is orthotopic heart transplantation. In this case, the work up should include a total body positron emission tomography and CT scans to exclude distant metastases [6]. Thus, the adequate multimodal treatment strategy should be discussed in a pluridisciplinary meeting in order to improve survival. Prognosis is poor. Nevertheless, it seems that for patients who survived the initial surgery, the prospect for long-term survival is very promising. The present case has been discussed in view of rarity of long-term survival in cardiac leiomyosarcomas since our patient is the longest survivor reported thus far in the English language medical literature with good interim quality of life (Table 1).

**Table 1:** **Cases of cardiac leiomyosarcoma reported in the literature with available data of treatment modalities and survival**

Reference	Age/sex	Origin site	Tumor size	Grade	Margin status	Treatment received	Survival (months)
[7]	64/F	Left atrium	17	3	Positive	Curative surgery and adjuvant chemotherapy	9
[7]	54/M	Right atrium	6	3	Positive	Curative surgery and adjuvant chemotherapy	13
[5]	45/F	Left atrium	10	3	Positive	Palliative surgery	5 days
[8]	46/M	Left ventricle	–	2–3	–	Palliative chemotherapy	13
[8]	42/F	Left ventricle	–	3	–	Palliative chemotherapy	18
[9]	63/F	Right ventricle	4			Curative surgery	36
[10]	86/F	Left atrium	4	–	–	Curative surgery	15
[11]	74/M	Left atrium	–	–	Positive	Curative surgery and adjuvant radiotherapy	8
[12]	54/F	Right atrium				Curative surgery	Few hours
[13]	35/F	Right atrium	5.6	3	Positive	Surgery and palliative chemotherapy	10

## Consent

Oral informed consent was obtained from the patient for publication of this case report and any accompanying images.
